# P53 regulates the migration of mesenchymal stromal cells in response to the tumor microenvironment through both CXCL12-dependent and -independent mechanisms

**DOI:** 10.3892/ijo.2013.2109

**Published:** 2013-09-23

**Authors:** SIANG-YO LIN, SONIA C. DOLFI, SOHRAB AMIRI, JAIDONG LI, TULIN BUDAK-ALPDOGAN, KUO-CHIEH LEE, CHRISTOPHER DERENZO, DEBABRATA BANERJEE, JOHN GLOD

**Affiliations:** 1 Departments of Pediatrics, University of Medicine and Dentistry of New Jersey, New Brunswick, NJ 08903, USA; 2 Pharmacology, University of Medicine and Dentistry of New Jersey, New Brunswick, NJ 08903, USA; 3 Medicine, The Cancer Institute of New Jersey, Robert Wood Johnson Medical School, University of Medicine and Dentistry of New Jersey, New Brunswick, NJ 08903, USA

**Keywords:** CXCL12, stromal cell-derived factor 1, mesenchymal stromal cell, p53

## Abstract

Mesenchymal stromal cells (MSCs) are multipotent fibroblast-like cells located in the bone marrow that localize to areas of tissue damage including wounds and solid tumors. Within the tumor microenvironment, MSCs adopt the phenotype of carcinoma-associated fibroblasts (CAFs) and stimulate tumor growth. Production of the chemokine CXCL12, also known as stromal cell-derived factor 1 (SDF-1), by MSCs is required for their *in vitro* migration in response to tumor cells and has also been implicated in stimulation of tumor growth. The tumor suppressor p53 regulates cellular migration, CXCL12 production and the promotion of tumor growth by carcinoma-associated fibroblasts (CAFs). We investigated the role of p53 in MSC migration to tumors. P53 inhibits the migration of MSCs in response to tumor cells in conjunction with a decrease in CXCL12 transcription. Conversely, decreased p53 activity leads to enhanced MSC migration. Interestingly, increased p53 activity inhibits MSC migration even in the context of high concentrations of exogenous CXCL12. These data show that stromal p53 status impacts the recruitment of MSCs to solid tumors through both regulation of CXCL12 production as well as other mechanisms. Stromal p53 may influence other important aspects of tumor biology such as tumor growth and metastasis through mechanisms distinct from CXCL12.

## Introduction

Mesenchymal stromal cells (MSCs) are multipotent cells that can be mobilized from the bone marrow and other tissues and localize to sites of inflammation including tumors and areas of injury (1–11). They have the capacity to differentiate into mesenchymal cell types including adipocytes, chondrocytes and myocytes (5). Upon incorporation into the stroma, MSCs promote tumor growth and metastasis (11,12). The molecular mechanisms regulating the mobilization and homing of MSCs to tumors have not been completely defined.

p53 is a transcription factor and tumor suppressor gene that is mutated in >50% of human cancers (13). It regulates a diverse array of cellular processes including cell cycle control, DNA repair, growth factor production and cell motility. Recent studies have suggested a role for stromal p53 in tumor biology. P53-null stromal fibroblasts promote more efficient tumor growth in a murine prostate cancer model (14) and there is a reduced latency of tumor formation in xenografts in p53-deficient mice compared to animals with wild-type p53 (15). Additionally, administration of MSCs carrying a p53 mutation decreased the time required to develop mammary tumors in *Apc^Min/+^Rag^2−/−^* mice (16). These observations were extended to human cancers where loss of heterozygosity in the TP53 gene in carcinoma-associated fibroblasts was identified in a number of tumor types (17–21). In experimental systems, tumors with decreased stromal p53 activity are more resistant to chemotherapy (22) and have increased tumor growth (14).

*In vitro*, tumor cells stimulate MSC motility and, over time, induce MSCs to adopt a carcinoma-associated fibroblast phenotype (9,12). Because p53 activity impacts cell mobility in fibroblasts (23,24) and may also play a role in the differentiation of mesenchymal cells (25) it is reasonable to suggest that p53 status influences the interaction between neoplastic and stromal cells. One mechanism for p53 regulation of tumor/stromal interaction is through modulation of CXCL12 production. Induction of MSC migration by tumor cells and stimulation of tumor growth by fibroblasts are dependent on stromal CXCL12 production (9,14).

Our data show that p53 regulates MSC motility. Increased p53 levels inhibit MSC mobility in response to tumor cells. The influence of p53 on MSC motility may be mediated, in part, through the transcriptional regulation of CXCL12. Conversely, MSCs with p53 knock-down show increased migration to tumor cells *in vitro* as well as to *in vivo* tumors. Interestingly, MSCs with increased p53 do not show increased migration in response to tumor conditioned media, even in an environment with a high concentration of exogenous CXCL12. This suggests that p53 is involved in other aspects of tumor/stromal interaction distinct from CXCL12 production. Our study demonstrates that stromal p53 status influences important aspects of the response of MSCs to tumor cells and provides additional insight into the molecular systems that regulate this interaction.

## Materials and methods

### Reagents and cell lines

C57BL/6J p53^−/−^ mice were generously provided by Dr Arnold Levine. C57BL/6J wt mice were purchased from Charles River Laboratories (Wilmington, MA, USA). Nude mice were purchased from Taconic Farms (Hudson, NY, USA). All animal procedures were approved by the Animal Care and Use Committee of RWJMS. MDA- MB231 cells were obtained from American Type Culture Collection (Manassas, VA, USA http://www.atcc.org); pooled human MSCs were obtained from Lonza (Walkersville, MD, USA, http://www.lonza.com) and used in early passage (below passage 8). MDA-MB231 cells were cultured in DMEM (Invitrogen, Carlsbad, CA, USA) supplemented with 10% fetal bovine serum (FBS) and 100 U/ml penicillin G and 100 *μ*g/ml streptomycin at 37°C in 5% CO_2_. Human MSCs were expanded in MesenCult media with hMSC stimulatory supplements (Lonza) and 10% FBS. Antibodies used in these studies included p53 (SC263) (Santa Cruz Biotechnology, Santa Cruz, CA, USA); α-tubulin and p21 (Sigma-Aldrich, St. Louis, MO, USA); GAPDH (Trevigen, Gaithersberg, MD, USA).

### Transwell chamber migration assay

A Falcon cell culture insert system along with companion 24-well tissue culture plate was used for the chemotaxis assay as described previously (9). The polyethylene terepthalate membrane, pore size 8 *μ*M, was selected to allow passage of mammalian cells. The insert was removed aseptically and placed in the notch of each well using forceps. MSCs (1–2×10^4^) were plated in 500 *μ*l of α-MEM (Invitrogen) supplemented with 10% heat-inactivated FBS and 1% penicillin/streptomycin, placed in the insert (top chamber). The bottom chamber contained either conditioned medium from tumor cells or control medium (DMEM containing 2% FBS and penicillin/streptomycin). Migration assays were stopped after 16 h and cells remaining on the top of the membrane were removed with a wet cotton swab. MCSs that had migrated through the membrane were stained with crystal violet. Stained cells were counted under high power magnification (×40). For some experiments Nutlin-3 (Sigma-Aldrich, St. Louis, MO, USA) or recombinant CXCL12 (R&D Systems, Minneapolis, MN, USA) were added to both the upper and lower chambers to the indicated final concentrations.

### Knockdown of p53 and CXCL12 in hMSCs using siRNA and lentiviral short hairpin RNA

Human MSCs (1.5×10^5^ cells) were plated in α-MEM (Invitrogen) with 10% FBS. After overnight incubation hMSCs were transfected with small interfering RNA (siRNA) specifically targeting p53 or CXCL12 or a scrambled sequence serving as a control (Thermo Scientific) using Lipofectamine 2000 (Invitrogen). Experiments were performed 2 days after transfection. Expression constructs containing short hairpin RNA (shRNA) sequences targeting p53 were obtained from Santa Cruz. hMSCs were infected using the manufacturer’s protocols and the knockdown of p53 was confirmed by western blotting. Cells were allowed to recover for 24 h prior to performing experiments.

### Production of conditioned medium from MDA-MB-231 cells

To obtain tumor conditioned medium, a ratio of 7.5×10^4^ tumor cells/700 *μ*l of DMEM containing 2% heat-inactivated FBS and 1% penicillin/streptomycin (Invitrogen) were incubated overnight. Conditioned medium was collected and spun down at 1,200 rpm to remove cellular debris. Supernatant was filtered through a 0.45-*μ*m steriflip filter (Millipore, Billerica, MA, USA) prior to use in experiments.

### Western blotting

Human MSCs were cultured to 80% confluence in a 150-mm tissue culture dish. Cells were then treated with MDA-MB231 conditioned medium or control medium with 25 *μ*M Nutlin-3 or vehicle [dimethyl sulfoxide (Sigma-Aldrich)] for 6 and 24 h. Following this treatment, cells were scraped from the dish and re-suspended in 200 *μ*l of radio-immunoprecipitation assay (RIPA) buffer plus 10 *μ*g/ml aprotinin, 10 *μ*g/ml leupeptin and 1 mM phenylmethylsulfonyl fluoride (PMSF). After clearing the cell lysates by centrifugation (16,000 g, 20 min at 4°C), the protein concentration was determined (Pierce, IL, USA). After boiling for 10 min, lysates (20 *μ*g) were resolved by SDS-polyacrylamide gel electrophoresis, transferred onto PDVF membrane (Millipore) and visualized by immunoblotting with antibodies of interest.

### Quantitative reverse transcription-PCR for SDF-1

Human MSCs were treated with Nutlin-3 as described above. Cells were then collected and RNA was extracted from MSCs using the RNeasy Mini kit (Qiagen Inc., Valencia, CA, USA) following standard procedures and quantified using the NanoDrop (Thermo Fisher Scientific, Rockford, IL, USA). Messenger RNA (mRNA) was used to generate complementary DNA (cDNA) which was amplified with a one-step RT-PCR kit (Applied Biosystems Inc., Foster City, CA, USA) using the MX4000 Multiplex Quantitative PCR System (Stratagene, Cedar Creek, TX, USA). CXCL12 cDNA was amplified by a CXCL12 taqman gene expression assay (Hs00930455, Applied Biosystems Inc., Foster City, CA, USA) using 100 ng of total RNA as starting material. 18s rRNA was amplified by the internal control 18s rRNA taqman assay (Applied Biosystems Inc.) using 1 ng of total RNA. Each RNA sample was assayed in quadruplicate and relative cDNA levels were determined after normalization to the internal 18s rRNA control.

### In vivo migration of MSCs to tumors

MSCs were isolated from the bone marrow of C57BL/6J p53^−/−^ mice as previously described (9). Briefly, mice were euthanized using bottled CO_2_ inhalation and the bilateral femurs were dissected out using sterile technique. The femurs were washed in phosphate-buffered saline containing 2% fetal bovine serum (FBS). Bone marrow cells were then obtained by flushing the femurs with PBS with 2% FBS. Cells were then filtered through a 70-*μ*m nylon mesh and plated in α-MEM with 10% FBS and penicillin/streptomycin and cultured for seven days and then used in experiments. For use as a control, murine MSCs were transfected with wild-type murine p53. The p53 wild-type (wt) expression plasmid was the generous gift of Dr Arnold Levine. The p53-wt plasmid has been previously described and encodes murine p53 and G418 resistance with an SV40 origin of replication (26). Wild-type MSCs were labeled using carboxyfluorescein diacetate succinimidyl ester (CFSE) (Invitrogen) and p53 knockout MSCs were labeled using CellTracker CM-Dil (Invitrogen) according to the manufacturer’s instructions. The breast cancer cell line MDA-MB-231 (American Type Culture Collection) was used in this study. Cells (106) along with Matrigel (50 *μ*l per injection; BD Biosciences) were injected subcutaneously into nude mice and tumors were allowed to reach a size of ∼5 mm in diameter. At this point, CFSE-labeled murine MSCs expressing human p53 and CM-Dil-labeled murine p53^−/−^ MSCs were mixed at a ratio of 1:1. Mixed MSCs (5×10^6^) were then injected subcutaneously 10 mm from each tumor. Seven days later the mice were sacrificed using CO_2_ inhalation. Tumors were dissected from the mice and a single cell suspension of each tumor was made. Tumors were dissected into small pieces using a scalpel and dissociated using collagenase (Roche, Mannheim, Germany) (0.035% wt/vol) in α-MEM at 37°C for 1 h. Tissue was further dissociated by pipetting several times through a 5cc pipette and debris was removed using a nylon strainer. The single cell suspension was them washed with α-MEM with 10% FBS. The cellular component of each tumor was then analyzed using flow cytometry.

### Statistical analysis

At least three independent experiments were performed for each *in vitro* migration assay. Results are presented as the means ± standard deviation. Statistical significance was determined using the Student’s t-test and a value of P<0.05 was considered statistically significant. Microsoft Excel software was use for statistical analysis.

## Results

### Regulation of MSC migration by p53

**Figure 1.**
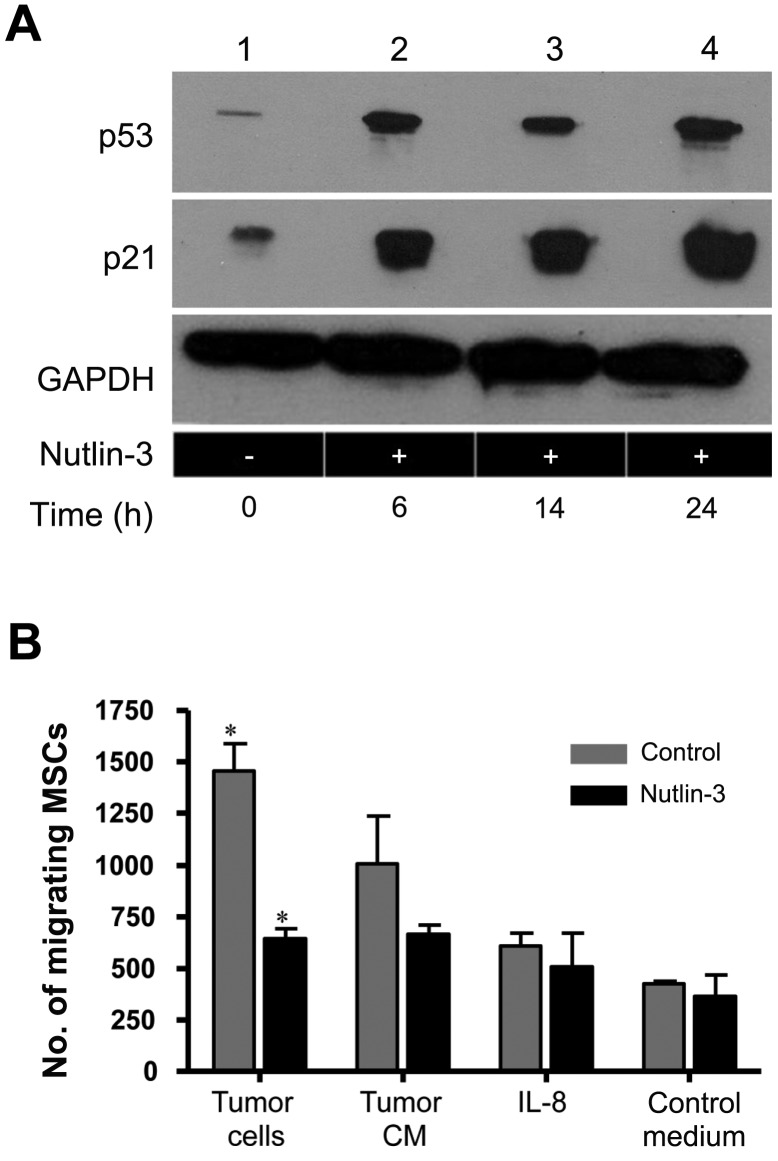

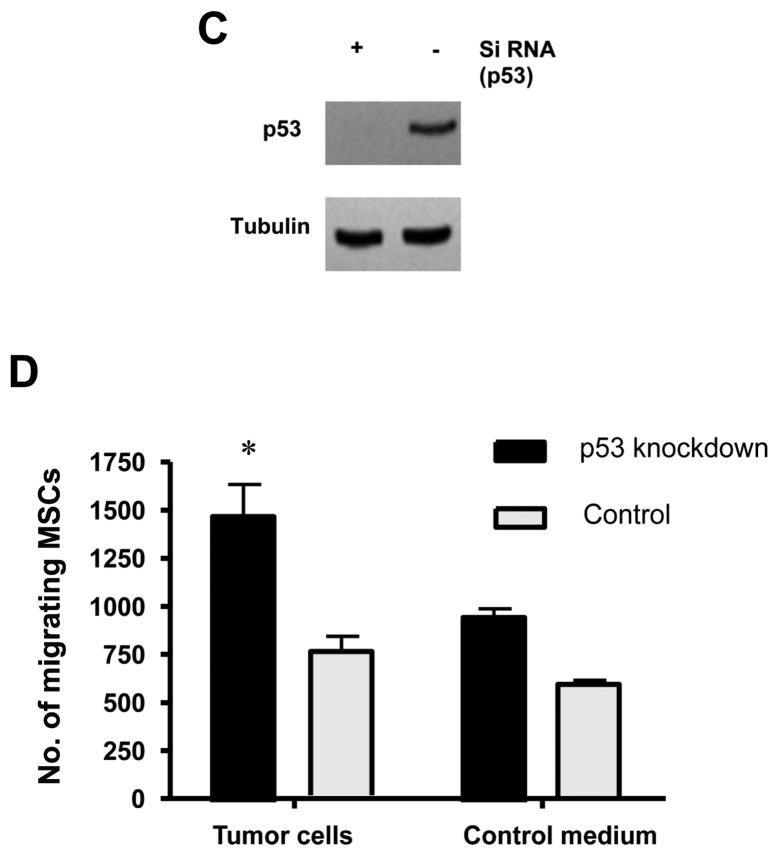
p53 regulates migration of MSCs in response to tumor cells. Human MSCs were treated with 25 *μ*M Nutlin-3 or vehicle (DMSO). (A) Nutlin-3 treatment led to increased levels of p53 protein as well as the p53 target p21 as assessed by western blotting. (B) Nutlin-3 treatment decreased hMSC migration in response to MDA-MB-231 cells (^*^P<0.001) and caused a non-significant decrease in hMSC migration in response to tumor cell conditioned medium. Nutlin-3 treatment did not change the migration of hMSCs in response to 10 ng/ml recombinant IL-8 (a known chemotactic stimulus for MSCs) or control medium. (C) Targeted siRNA was used to knockdown expression of p53 in hMSCs as demonstrated by western blot analysis of cell lysate. (D) Decreased levels of p53 led to increased MSC migration in response to tumor cells as well as to control medium (^*^P<0.0037 compared to si-RNA control).

Human MSCs were treated with the murine double minute 2 (MDM2) antagonist, Nutlin-3, leading to expected increases in p53 as well as increases in the p53 target p21 (Fig. 1A). In conjunction with increased p53 levels, the *in vitro* migration of MSCs in response to MDA-MB-231 tumor cells was decreased. The migration to tumor conditioned media showed a non-significant trend toward decreasing and Nutlin-3 did not inhibit the migration of MSCs in response to interleukin 8 (IL-8) (Fig. 1B). There was minimal basal migration of MSCs in response to control medium and this was not changed by treatment with Nutlin-3. When levels of p53 were decreased using siRNA (Fig. 1C), MSCs exhibited increased migration in response to tumor cells (Fig. 1D). These results suggested that p53 plays a role in regulating the response of MSCs to tumor cells.

### Exposure to tumor-conditioned medium does not influence MSC p53 activity

**Figure 2.**
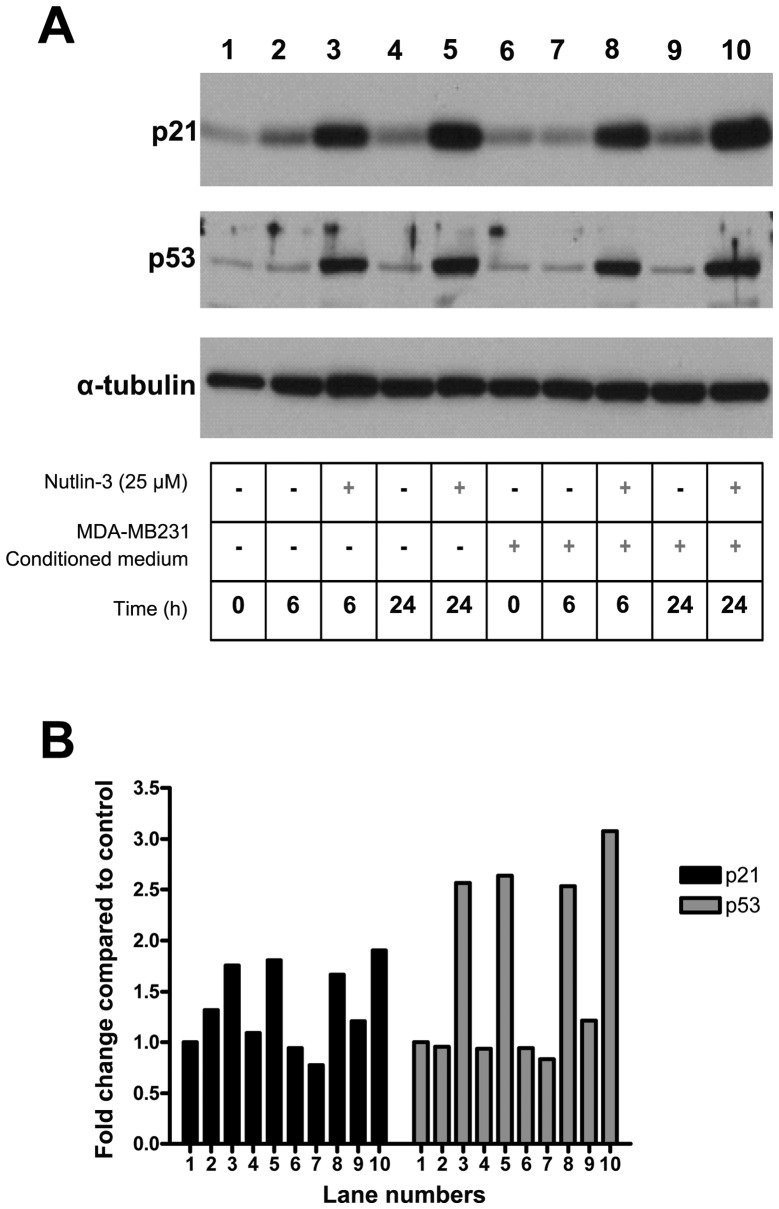
Treatment with tumor conditioned medium does not affect MSC p53 levels. hMSCs were treated with MDA-MB-231 conditioned medium or control medium with or without 25 *μ*M Nutlin-3. A, Western blot analysis of protein extracts collected over 24 h revealed that tumor conditioned medium alone did not influence MSC p53 or p21 levels. B, Treatment with Nutlin-3 led to increased p53 activity in the presence of both control medium and tumor-conditioned medium.

hMSCs were exposed to a combination of Nutlin-3 and MDA-MB-231 conditioned medium. As expected, exposure of MSCs to nutlin-3 led to increased levels of p53 as well as its target p21. However, exposure of MSCs to tumor conditioned medium did not impact p53 levels (Fig. 2). This result indicates that, while the p53 level influences MSC migration in response to tumor cells, induction of MSC motility by TCM is not mediated by changes in p53 activity.

### Increased p53 level leads to decreased CXCL12 production by MSCs

**Figure 3.**
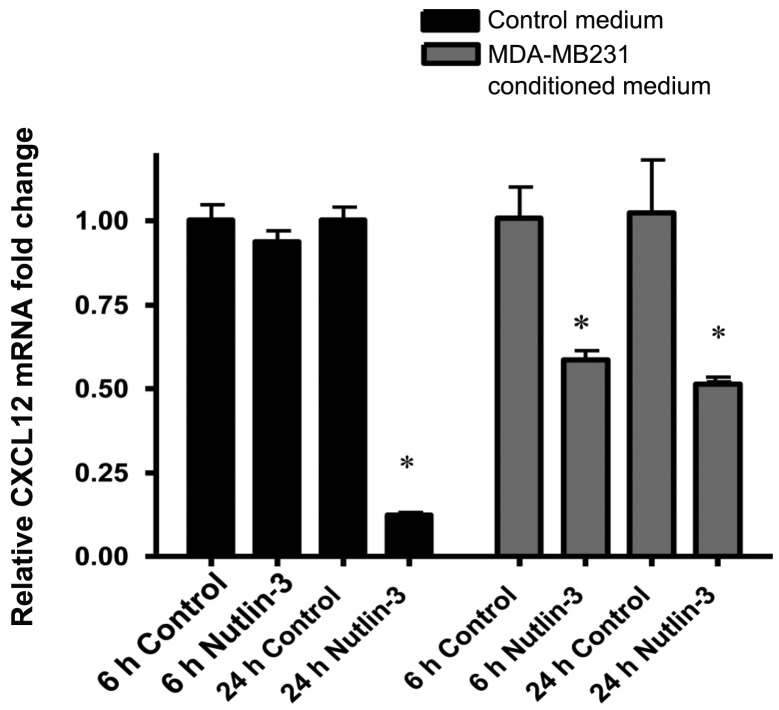
Nutlin-3 decreases CXCL12 mRNA levels in hMSCs. Human MSCs were treated with control medium or tumor conditioned medium with or without 25 *μ*M Nutlin-3. Q-RT-PCR of the extracted RNA after 24 h of Nutlin-3 treatment revealed decreased CXCL12 mRNA levels in MSCs cultured in control medium. Decreased CXCL12 mRNA levels were seen in hMSCs cultured in tumor-conditioned medium at both 6 and 24 h (^*^P<0.04).

We next explored the mechanism of regulation of MSC migration by p53. Because production of CXCL12 by MSCs is required for their migration in response to tumor cells (9), we investigated the effect of increased p53 on CXCL12 production by MSCs. Nutlin-3 treatment decreased hMSC CXCL12 mRNA levels after 24 h. CXCL12 mRNA levels in MSCs exposed to conditioned medium from MDA-MB-231 cells were decreased by nutlin-3 treatment at both 6- and 24-h time intervals (Fig. 3). These data suggested that p53 impacts MSC migration through regulation of CXCL12 transcription.

### The increased motility of MSCs due to p53 knockdown is dependent on CXCL12

**Figure 4.**
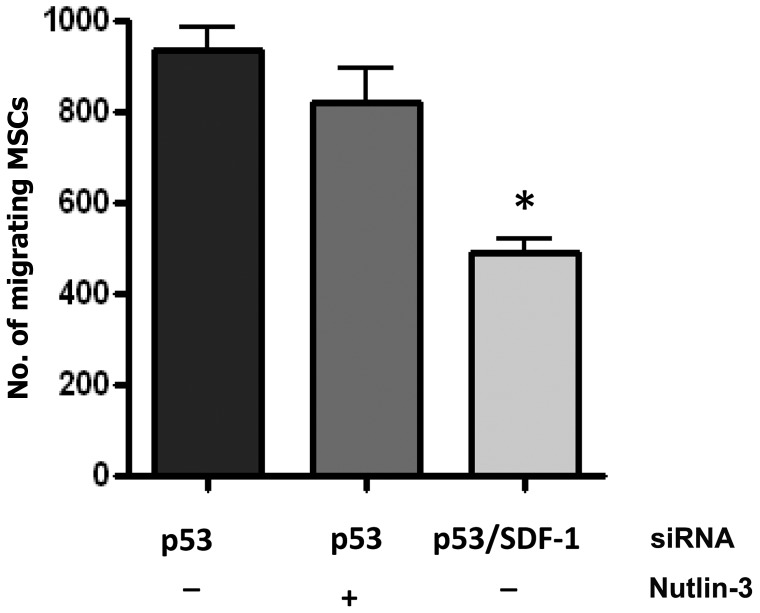
Enhanced MSC motility due to decreased p53 level is dependent on CXCL12. Human MSCs with siRNA-mediated p53 knockdown demonstrated enhanced migration in response to MDA-MB-231 cells. Treatment with 25 *μ*M Nutlin-3 did not cause a decrease in migration. Knockdown of CXCL12 production using siRNA decreased the migration of p53^−/−^ MSCs suggesting that decreased p53 activity leads to increased MSC mobility through increased CXCL12 transcription. (n=4) (^*^P= 0.007).

To further demonstrate the mechanism of action for Nutlin-3 inhibition of MSC chemokinesis, MSCs with p53-knockdown were treated with Nutlin-3. Nutlin-3 did not diminish the migration of MSCs with p53 knockdown induced by tumor cells (Fig. 4), indicating that p53 is required for Nutlin-3-mediated suppression of migration.

In order to determine whether the regulation of CXCL12 plays a role in the increased motility of MSCs with p53 knockdown, MSCs with both p53 and CXCL12 knockdown were generated using siRNA. CXCL12 knockdown blocked the increased motility of MSCs with p53 knockdown (Fig. 4).

### P53 regulates MSC migration using multiple mechanisms

**Figure 5.**
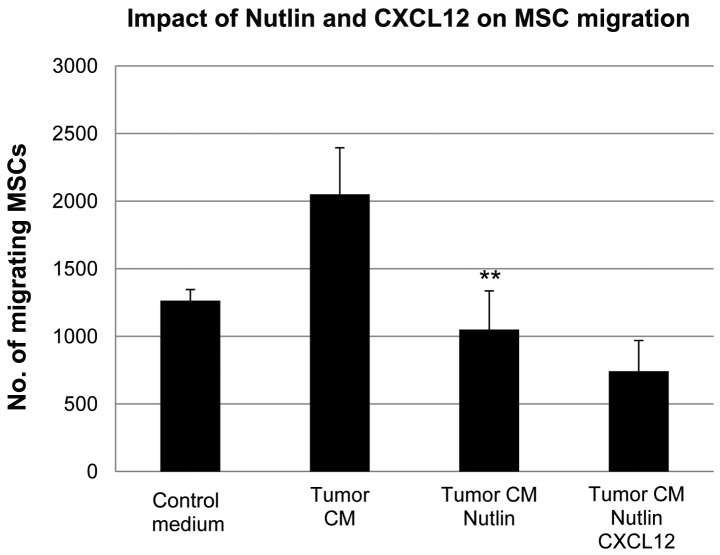
Decreased MSC motility in response to increased p53 activity is not reversed by CXCL12. MSCs were treated with 25 *μ*M Nutlin-3. Recombinant CXCL12 was added to both the upper and lower chambers of the Boyden chamber and migration of MSCs in response to MDA-MB-231 cell conditioned media was then allowed to proceed overnight. Exogenous CXCL12 did not reverse the decreased migration of MSCs observed with Nutlin-3 treatment. ^**^Migration of MSCs in response to Tumor CM was significantly decreased in the presence of Nutlin-3 (P<0.005).

We then sought to determine whether the effect of p53 levels on MSC migration was exclusively through its role in the regulation of CXCL12 production. Recombinant CXCL12 protein was added to human MSCs that had been treated with Nutlin-3. While exogenous application of CXCL12 does stimulate migration of MSCs in response to tumor conditioned medium (9), recombinant CXCL12 failed to increase migration of MSCs treated with Nutlin-3 (Fig. 5). This result suggests that, in addition to regulating the production of CXCL12, p53 may impact MSC migration through additional mechanisms.

### The in vivo homing capability of p53-null MSCs to tumors is enhanced compared to wild-type

**Figure 6.**
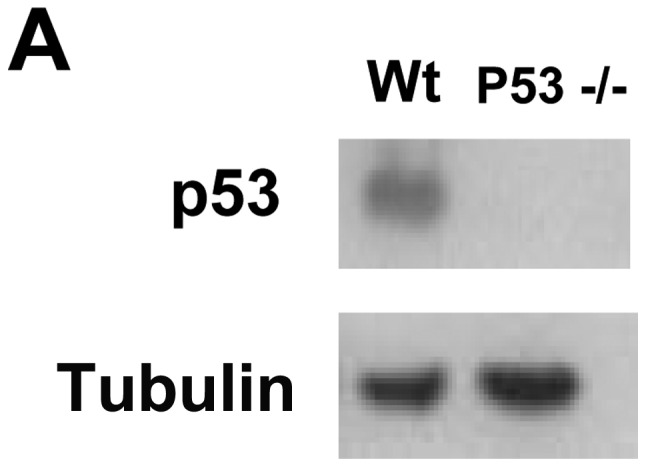

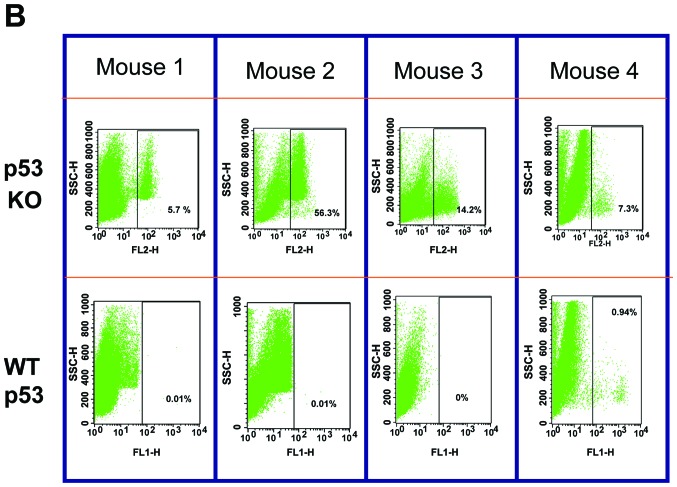
MSCs with p53 knock-down localize more efficiently to tumors *in vivo* than MSCs with wild-type p53. Murine MSCs were isolated from C57BL/6J p53^−/−^ mice. In order to generate cells with functional p53, murine p53^−/−^ cells were transfected with a plasmid encoding wild-type murine p53. (A) Western blot analysis of protein isolated from p53^−/−^ murine cells and p53-transfected murine cells shows expression of human p53. (B) MSCs expressing wt p53 and p53^−/−^ MSCs were differentially labeled using green (CFSE) and red (CM-DiI) fluorescent dyes, respectively. The cells were combined in a ratio of 1:1 and injected subcutaneously 5 mm from established MDA-MB-231 tumors in nude mice. Three days after injection the animals were sacrificed and tumors were harvested. Single cell suspensions were made from the tumors and the percentages of both p53 knockdown and wild-type MSCs were determined using flow cytometry. An increased percentage of labeled p53 knockdown MSCs were present in the tumors compared to wild-type MSCs. These data indicate that decreased p53 levels lead to increased chemokinesis of MSCs in response to tumors.

To determine whether p53 regulates homing of MSCs to tumor sites *in vivo*, we used bone marrow-derived MSCs isolated from p53-null mice. The human wild-type p53 gene was introduced into the MSCs using a lentiviral vector. Western blot analysis demonstrated that MSCs isolated from p53-null animals were deficient in p53 protein and that after transfection of wild-type p53 gene, the expression of the tumor suppressor was detected (Fig. 6A). Wild-type MSCs were labeled using CFSE and p53 knockout MSCs were labeled using CM-Dil. Wild-type and p53^−/−^ MSCs were mixed in a 1:1 ratio and subcutaneously co-injected into nude mice 5 mm from established tumors (MDA-MB231). At day 7 after administration of the MSCs, animals were sacrificed and tumors were collected and used to generate single cell suspensions. The ratio of wild-type to p53^−/−^ cells in the tumor was then determined using flow cytometry. Increased number of p53-null MSCs were found in the tumors compared to wild-type MSCs, indicating that the *in vivo* homing capability of MSCs was enhanced in cells with decreased p53 activity (Fig. 6B).

## Discussion

Carcinoma associated fibroblasts are known as a key mediator of tumor growth and progression. A better understanding of signaling pathways underlying communication between neoplastic cells and MSCs is important to better define their role in tumor biology. MSCs are mobilized from bone marrow and other tissues and integrate into the tumor stroma (2,7,9,12). They impact diverse aspects of tumor progression such as angiogenesis, tumor growth and metastasis (11,12,27). While there are likely to be multiple mechanisms for the intercellular signaling between MSCs and tumor cells, the chemokine, CXCL12 has been implicated in MSC chemotaxis and homing to tumors, MSC-mediated stimulation of tumor growth and cellular tissue invasion (9,14,27,28).

Recently, Addadi and colleagues demonstrated that CXCL12 production is downregulated in tumor stomal fibroblasts by p53 and that this change in the stroma has a significant impact on tumor growth (14). Importantly, it has also been shown that systemically delivered p53-deficient MSCs decrease tumor latency (14,16), providing further evidence that the stromal p53 status is important in tumor growth.

Clinical studies have reinforced the potential role of p53-mediated signaling in tumor/stromal interactions. TP53 mutations have been reported within the stroma of sporadic breast cancers (19) and both TP53 mutation as well as p53 expression in stromal fibroblasts are associated with lymph node metastasis in breast cancer (19,29). Others have found that changes in the stromal expression of the p53 target, p21, is associated with increasing malignancy in breast cancers as well as an increased growth rate of human breast cancer xenografts when tumors are implanted along with p21 deficient fibroblasts (30). Interaction with cancer cells can influence the p53 status of fibroblasts. Co-culture with the small cell lung cancer cell line H1299 inhibits the induction of p53 expression by cisplatin (31). However, in alignment with our data, there was no change in basal p53 levels. These data suggest that p53-dependent pathways play an important role in tumor stromal biology.

Our experiments build on this study and demonstrate that additional functional consequences of the p53 status of MSCs include changes in migration efficiency both *in vitro* and *in vivo*. Our data also suggest that increased CXCL12 production is not the only important outcome of decreased p53 function in stromal cells. Even in the context of exogenous CXCL12, increased p53 levels lead to impaired motility of MSCs in response to tumor cells. It is likely that the increased rates of tumor formation and progression due to aberrant stromal p53 are a consequence not only of increased CXCL12, but of multiple changes in the stroma. The mechanisms of p53-mediated regulation of CXCL12 expression as well as identification of other important targets of p53 within the tumor stroma are important areas of continued investigation.

In conclusion, the complex interplay between tumor cells and the surrounding non-neoplastic cellular components of solid tumors remains incompletely understood. This study suggests that stromal p53 is a critical mediator of this interaction through multiple pathways. Stromal p53 status influences not only CXCL12 signaling with tumor stromal cells, but also impacts the stromal response to neoplastic cells through other mechanisms.
